# How to perform a root cause analysis for workup and future prevention of medical errors: a review

**DOI:** 10.1186/s13037-016-0107-8

**Published:** 2016-09-21

**Authors:** Ryan Charles, Brandon Hood, Joseph M. Derosier, John W. Gosbee, Ying Li, Michelle S. Caird, J. Sybil Biermann, Mark E. Hake

**Affiliations:** 1 Department of Orthopaedic Surgery, University of Michigan, 2912 Taubman Center, SPC 5328, 1500 E. Medical Center Dr., Ann Arbor, MI 48109 USA; 2 Center for Healthcare Engineering & Patient Safety, College of Engineering, University of Michigan, Ann Arbor, MI USA; 3 Department of Internal Medicine, University of Michigan, Ann Arbor, MI USA; 4 Department of Biomedical Engineering, University of Michigan, Ann Arbor, MI USA

**Keywords:** Resident education, Root cause analysis, Medical errors, Quality improvement, Adverse events, Patient safety

## Abstract

Providing quality patient care is a basic tenant of medical and surgical practice. Multiple orthopaedic programs, including The Patient Safety Committee of the American Academy of Orthopaedic Surgeons (AAOS), have been implemented to measure quality of surgical care, as well as reduce the incidence of medical errors. Structured Root Cause Analysis (RCA) has become a recent area of interest and, if performed thoroughly, has been shown to reduce surgical errors across many subspecialties. There is a paucity of literature on how the process of a RCA can be effectively implemented. The current review was designed to provide a structured approach on how to conduct a formal root cause analysis. Utilization of this methodology may be effective in the prevention of medical errors.

## Background

Quality of care has been an evolving area of interest in both medical and surgical specialties. Ensuring appropriate, efficient, effective and quality care is now a regulated branch of medical practice. Organizations like the National Surgical Quality Improvement Program measure the quality of surgical care and encourage hospitals to implement formal quality improvement projects [1]. Furthermore, Medicare has stopped providing reimbursement for complications deemed as “preventable” [2]. Preventable orthopaedic complications can include wrong-site surgery and preoperative deficiencies resulting in postoperative complications such as surgical site infections, catheter-associated urinary tract infections, and venous thromboembolism [3]. As such, both hospitals and payors have new incentives to reduce surgical complication rates. Multiple orthopaedic programs, including the Patient Safety Committee of the American Academy of Orthopaedic Surgeons (AAOS), have been developed to improve patient safety on national, state, and local levels. The Patient Safety Committee supports numerous healthcare agencies to improve healthcare quality and reduce medical errors [4]. The Joint Commission now expects physicians to develop integrated patient safety systems including sentinel event reviews and Root Cause Analysis. The purpose of this paper is to present a model using Root Cause Analysis (RCA) as an effective and efficient means of promoting patient safety as a complement to a department or health system patient safety structure.

### Root cause analysis

RCA is a systematic approach aimed at discovering the causes of close calls and adverse events for the purpose of identifying preventative measures [5]. RCA teams look beyond human error to identify system issues that contributed to or resulted in the close call or adverse event [6]. The goal is to answer what happened, why did it happen, and what can be done to prevent it from happening again? [7, 8]. The process includes document reviews and interviews with the parties involved in the event. Flow diagramming, cause and effect diagramming, and identifying root causes and contributing factors help to organize the events and determine why an error occurred. Based on the root causes and contributing factors, actions can be developed to prevent the error from recurring. Measuring the outcome of an intervention is also planned in order to determine the success of the RCA. Tools to assist the team include triggering questions, the five rules of causation, and action hierarchy [7].

### RCA process

The goal of performing an RCA is to protect patients by identifying and changing factors within the healthcare system that can potentially lead to harm. There are 9 steps (Table 1) which serve as a guide for performing an effective RCA. Before a RCA can begin, honest and open reporting of errors is required [9]. A Department should strongly encourage residents, midlevel providers, and faculty to report adverse events and close calls (or near misses). A risk based triaging system should be used to evaluate the report to determine if an RCA is required. At our institution, there is a patient care committee comprised of faculty and residents who review incident reports and decide if an event would benefit from an RCA. If an RCA is required, it would be assigned to a small team consisting of 4 to 6 individuals who have fundamental knowledge of the specific area involved [7]. Team members should consist of physicians, supervisors, ancillary staff and quality improvement experts. It is important that members of the RCA team are not involved in the case being reviewed to ensure objectivity [10, 11]. Time to completion of an RCA varies depending complexity of the case, time required to conduct interviews and synthesize information, and barriers to implementation of corrective actions; however, a typical investigation should range between one to three months.

**Table 1:** Process of root cause analysis (RCA)

Step 1: Identify Adverse Event • Honest and open reporting of adverse events • Committee review of clinical documentation to understand basics of what event happened? When? Who was involved? How and why did it happen? • Identify appropriate RCA investigations
Step 2: Organize a Team • Team should consist of 4–6 members of clinicians, supervisors, quality improvement experts with fundamental knowledge of specific area of interest • Ensure that despite members having different levels of authority, everyone should be treated as equals • Members should not be directly involved with the case in question • Appoint an unbiased team leader/facilitator
Step 3: Develop an Initial Flow Diagram • Use a flowchart to describe the processes leading to the event • Organizing the information to reach a mutual understanding of the problem
Step 4: Develop an Event Story Map • Use of Triggering questions to guide further investigation • Conduct thorough interviews with all parties involved in event • Thorough review of clinical documentation surrounding the event
Step 5: Develop a Cause and Effect Diagram • Identify a single problem statement • Identify Actions and Conditions that caused the problem statement • These categories should address communication problems, policies, rules, procedures and human errors leading to the event
Step 6: Identify Root Cause Contributing Factors (RCCF) • Describe how a cause led to an effect and increased the likelihood of adverse event • Apply 5 rules of causation for crafting RCCF statements
Step 7: Develop Corrective Actions • Identify barriers and risk reduction strategies to prevent root cause from recurring • Multiple actions may be required • Implement a trial test of corrective action
Step 8: Measure Outcomes • Develop outcome measurements to ensure appropriate implementation of actions • Track quantifiable data to document effectiveness of actions over time • Evaluate and fine-tune improvement efforts if needed
Step 9: Communicate Results • Communicate results of RCA to all staff involved in event and more broadly if applicable

The next step of the RCA process is to create an “initial flow diagram” depicting the known sequence of events leading up to the adverse event being investigated. The purpose of the initial flow diagram is to present the known facts and serve as a springboard to investigate what contributed to each event [12]. Development of a basic flow diagram facilitates a mutual understanding of the event and problem.

**Fig. 1:**
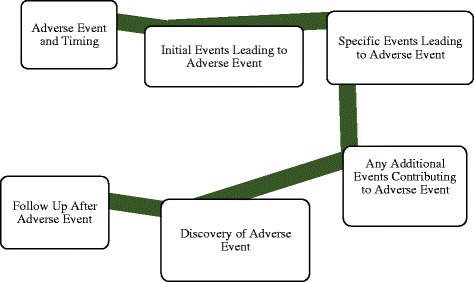
Event story map creation conveys significant detail of event after chart reviews and personnel interviews

An extensive list of “triggering questions” provides a clinical context and helps postulate what occurred during the time period in which the adverse event took place [13, 14]. Triggering questions serve as cognitive aids to identify areas of inquiry that may not have been previously considered. The questions cover communication, training, engineering, equipment, rules, policies, procedures, and barriers. To answer these questions, any individual who may have contributed to the progression of the adverse event is subsequently interviewed. This includes attending physicians, residents, mid level providers, nursing, engineering, and ancillary staff. The purpose of these questions and ensuing interviews is to identify exactly what occurred, and fill in details of the initial flow diagram, thus creating an “event story map” (Fig. 1). The event story map conveys in significant detail what happened and why it happened utilizing the information collected during the interview process.

**Fig. 2:**
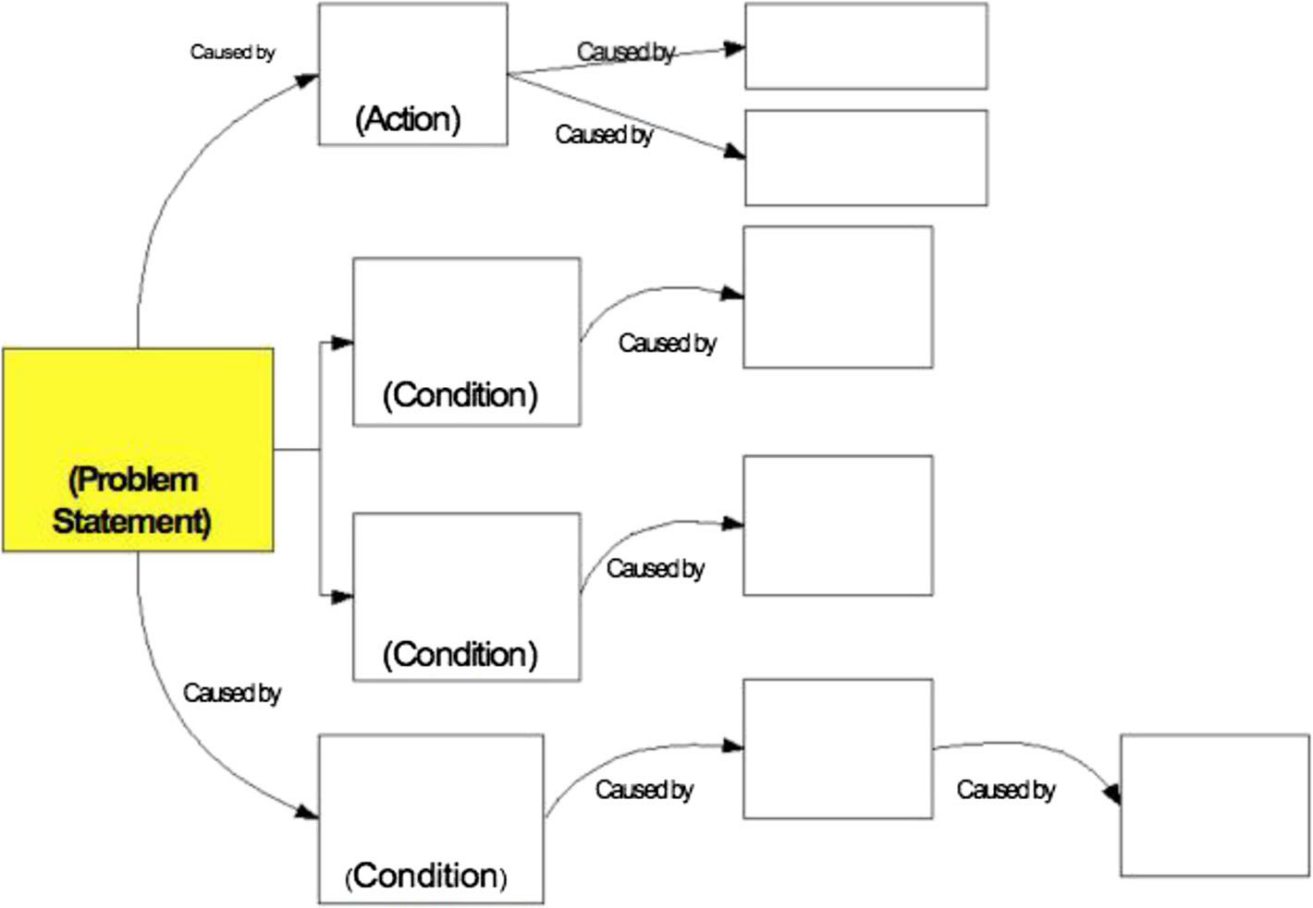
A Cause and Effect Diagram is read from left to right connected by “caused by” statements. From the cause and effect diagramming model in Apollo Root Cause Analysis by Dean L. Gano [15]

Once the Event Story Map is constructed, it is necessary to develop a “cause and effect” diagram. A cause and effect diagram is composed of a problem statement, an action, and two to three conditions [15]. These categories should address communication problems, policies, rules, procedures and human errors leading to the event. Each causal event box in the diagram is connected to the preceding box by a “caused by” statement (Fig. 2). This process is continued until knowledge of the event is exhausted, it becomes apparent that additional investigation is required, or the causal events identified are too far removed to be of value. The purpose of crafting a cause and effect diagram is to help the teams identify causal links and ascertain “root cause contributing factors” (RCCF) for each event.

Crafting a RCCF statement begins by describing how something (cause), led to something (effect), that increased the likelihood of an undesirable outcome (event) [14]. After the initial RCCF statement or statements are created, the “Five Rules of Causation” are applied to finalize each statement (Table 2) [16, 17]. By correctly crafting the RCCF statement, the teams’ findings are distilled into one or two sentences that describe what happened and why it is important to expend time and/or resources to correct it. This creates a road map leading to the development of corrective actions and their respective process or outcome measures. The implementation of these actions is what ultimately improves patient safety.

**Table 2:** Five rules of causation for root cause contribution factor

Five Rules of Causation	
1. Clearly show the cause and effect relationship.	
2. Use specific and accurate descriptors for what occurred, rather than negative and vague words.	
3. Human errors must have a preceding cause.	
4. Violations of procedure are not root causes, but must have a preceding cause.	
5. Failure to act is only causal when there is a pre-existing duty to act.	

The RCCFs are placed on the event story map before the primary event where there is a system vulnerability that should be addressed. This placement indicates the location where an existing barrier needs to be reinforced or where a new barrier needs to be created. Ideally there will be RCCFs identified at multiple points along the event story map, which graphically represents how care processes are designed to be fault-tolerant.

Finalizing an event story map with appropriately identified RCCF statements would be meaningless to patients if it did not lead to action and change. Using the RCCF statements, specific actions with the goal of sustained system improvement are implemented [12, 18]. While the implementation of the actions is left to department and hospital leadership, the RCA team is responsible for identifying an individual to follow the implementation process and confirm the changes have in fact been made. A properly crafted process or outcome measure should be specific, quantifiable, and provide a timeline on when it is going to assessed [19]. It should clearly tell you if the action that was implemented resulted in the desired system change. Finally, corrective actions identified throughout the RCA should be shared amongst appropriate parties not only involved in the RCA and adverse event or close call but also with other hospital staff and departments as a means to promote quality improvement [12, 19].

## Conclusion

Elimination of medical errors and promotion of patient safety through quality improvement programs continues to be an evolving area of interest. Payment schemes and national programs have been developed with the purpose of ensuring quality healthcare. However, the orthopaedic literature is sparse on how to effectively develop and implement quality improvement programs. Our model provides guidance on the development and implementation of quality improvement initiatives to reduce surgical errors.

## Abbreviations

RCA: Root cause analysis
RCCF: Root cause contributing factors
